# Reciprocal regulation of Abl kinase by Crk Y251 and Abi1 controls invasive phenotypes in glioblastoma

**DOI:** 10.18632/oncotarget.6096

**Published:** 2015-10-12

**Authors:** Sushil Kumar, Bin Lu, Updesh Dixit, Sajjad Hossain, Yongzhang Liu, Jing Li, Peter Hornbeck, Weiming Zheng, Adam G. Sowalsky, Leszek Kotula, Raymond B. Birge

**Affiliations:** ^1^ Department of Microbiology, Biochemistry and Molecular Genetics, Cancer Center, Rutgers New Jersey Medical School, Newark, New Jersey, USA; ^2^ Institute of Biophysics, School of Life Sciences, Wenzhou Medical University, Wenzhou, China; ^3^ Attardi Institute of Mitochondrial Biomedicine, School of Life Sciences, Wenzhou Medical University, Wenzhou, China; ^4^ Departments of Urology and Biochemistry and Molecular Biology, SUNY Upstate Medical University, New York, NY, USA; ^5^ Cell Signaling Technology, Danvers, MA, USA; ^6^ Department of Neurosurgery, The First Affiliated Hospital of Wenzhou Medical University, Wenzhou, China; ^7^ Department of Medicine, Beth Israel Deaconess Medical Center, Harvard Medical School, Boston, MA, USA

**Keywords:** non-canonical Crk signaling, Abi1, glioblastoma multiformae, cell invasion

## Abstract

Crk is the prototypical member of a class of Src homology 2 (SH2) and Src homology 3 (SH3) domain-containing adaptor proteins that positively regulate cell motility via the activation of Rac1 and, in certain tumor types such as GBM, can promote cell invasion and metastasis by mechanisms that are not well understood. Here we demonstrate that Crk, via its phosphorylation at Tyr251, promotes invasive behavior of tumor cells, is a prominent feature in GBM, and correlating with aggressive glioma grade IV staging and overall poor survival outcomes. At the molecular level, Tyr251 phosphorylation of Crk is negatively regulated by Abi1, which competes for Crk binding to Abl and attenuates Abl transactivation. Together, these results show that Crk and Abi1 have reciprocal biological effects and act as a molecular rheostat to control Abl activation and cell invasion. Finally, these data suggest that Crk Tyr251 phosphorylation regulate invasive cell phenotypes and may serve as a biomarker for aggressive GBM.

## INTRODUCTION

The CT10 regulator of kinase (Crk) family of adaptor proteins, CrkI and CrkII, and a related gene product CrkL, are versatile Src homology 2 (SH2) and Src homology 3 (SH3) domain-containing proteins that regulate protein-interactions downstream of tyrosine kinases [1-4]. The best understood mechanism for Crk signaling involves the recruitment of specific tyrosine phosphorylated proteins, such as paxillin [5] and p130cas [6], [7] via the Crk SH2 domain and recruitment of specific proline-rich proteins, such as the guanine nucleotide exchange factors (GNEFs) DOCK180 [8], [9] and C3G [10] to the N-terminal SH3 domain (SH3N). These coordinated protein assemblages in turn activate small GTPases [1], to control the actin cytoskeleton and promote cell motility and adhesion. In contrast, the ability of Crk to bind tyrosine phosphorylated proteins is negatively regulated by Tyr221 phosphorylation (Tyr207 in CrkL), which induces an intramolecular interaction between Tyr221 and the SH2 domain, thereby terminating the interaction between Crk and tyrosine phosphorylated proteins [11] [12].

Recently, we described a non-canonical pathway for Crk signaling whereby Tyr221 phosphorylation, rather than turning off Crk signaling, redirects signaling from the conventional SH2->SH3(N) circuitry to an unconventional SH3(N)->pSH3(C) circuitry, mediated by iterative tyrosine phosphorylation of Crk on Tyr221 and Tyr251 or Tyr239 [13]. Using bioinformatics, high throughput SH2 profiling, and co-immunoprecipitation we showed that Tyr239 and Tyr251, when phosphorylated, interact with C-terminal Src kinase (CSK) and Abl respectively, providing a link between Crk phosphorylation and the activation of non-receptor tyrosine kinases known to impinge on Crk biology [13]. In the case for Abl kinase, which induces non-canonical signaling by binding to Crk and promoting iterative phosphorylation on Tyr221, Tyr239, and Tyr251, the pTyr251 motif binds the Abl SH2 domain and induces Abl transactivation prior to the dissociation of Crk from Abl [13].

In contrast to Crk, other SH3 domain-containing adaptor proteins concomitantly interact with Abl but do so with opposing effect on Abl activity. For example, Abi1, an adaptor protein identified in screens for spectrin [14], Eps8 [15] and Abl kinase interacting proteins [16] is a negative regulator of Abl, which has been biochemically linked to the Abi1 SH3 domain [17-19]. In prostate cancer LnCap cells, overexpression of Abi1 Isoform 2 abrogates Abl kinase activity and decreases Tyr412 in the activation loop of kinase domain [19].

In addition to interacting with Abl, Abi1 contributes to the composition of the WAVE protein complex that regulates actin polymerization downstream from a variety of receptors [20], and upstream from Arp2/3 [21]. Abi1 also plays a critical role in WAVE complex stability and activity [22] via its direct interaction with Rac1 [23]. Indeed, the invasion phenotypes of several cancers are associated with dysregulation of WAVE complex, and both increased function of WAVE complex genes and gene deletions are found in tumors [24, 25]. Interestingly, Abi1 can either positively or negatively contributes to tumor grade and progression depending on tumor types [19], but whether this reflects interaction with Abl or WAVE components is not defined.

In the present study, we show that Crk and Abi1 have reciprocal and opposing effects on Abl kinase activity, whereby Abl is affirmatively regulated by pTyr251 Crk phosphorylation, and negatively regulated by Abi1 isoform 2. We demonstrate that Crk Tyr221 and Tyr251 are routinely and iteratively tyrosine phosphorylated by EGF in EGFR-expressing glioblastoma cell lines (GBM), and at both the biochemical and cell biological level, the level of Tyr251 Crk phosphorylation strongly correlates with Abl kinase activation, as well as the invasive properties of GBM cells. Moreover, in patient-derived clinical GBM samples, Crk expression and Y251 phosphorylation is upregulated, and Abi1 levels are downregulated, and the level of Crk and phospho Crk Y251 is inversely correlated with patient survival outcomes. These data support the idea that Abi1 has tumor suppressor-like properties in GBM, and does so by unleashing the Crk non-canonical phosphorylation axis to activate Abl kinase and induce invasive phenotypes.

## RESULTS

### Reciprocal expression of Crk and Abi1 in human GBM tumor samples

**Figure 1:**
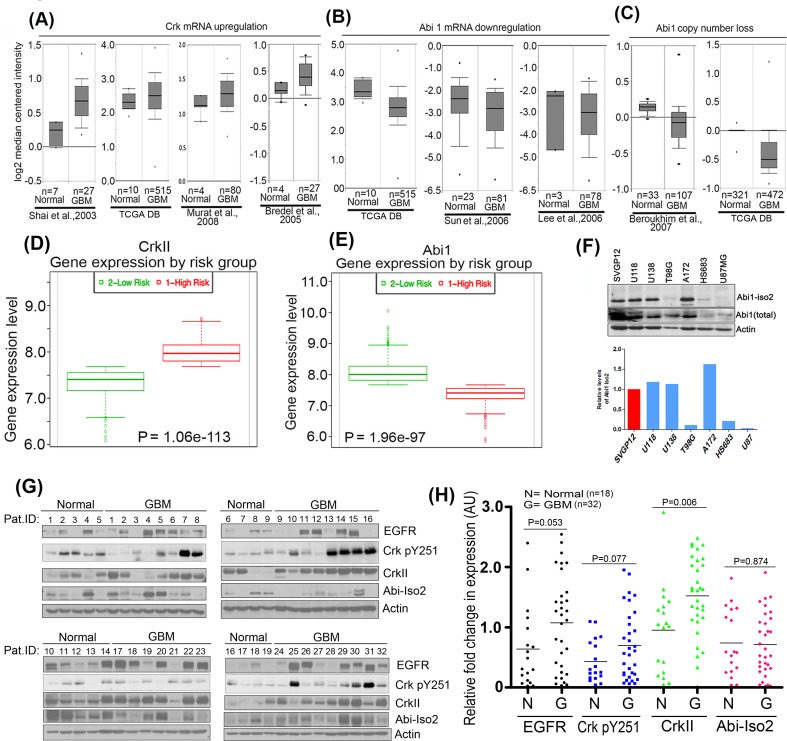
Reciprocal regulation of Crk and Abi1 genes in glioblastoma **A.** Bioinformatics meta-analysis of cancer genome sequencing data: **A.**-**C.** Oncomine database was used to analyze the genomic and transcriptomic variations in Crk and Abi1 genes by meta-analysis of indicated studies data. The result of the analysis is represented in box-whisker plots and copy numbers and mRNA expression values are presented on a log2 scale. The results indicate an upregulation of Crk gene expression **A.** downregulation of Abi1 gene expression **B.** suppression in copy number of Abi1 gene **C.** (*P* < 0.003). **D.**-**E.** SurvExpress analysis using TCGA Brain 2013 data to assess survival outcomes in risk groups as compared with gene expression profiles of Crk(D). and Abi1 (E). **F.** Western blot analysis validates bioinformatics analysis indicating a significant suppression in Abi1-Iso2 levels in T98G, HS683 and U87MG cells. **G.** Western blot analysis of patient GBM tissue and normal tissue samples: 18 normal and 32 GBM biopsied samples (obtained from Wenzhou University Medical Center) were immunoblotted with anti-EGFR, anti-CrkpY251, anti-Crk, or anti-Abi-Iso2 antibodies. **H.** Samples were normalized to the actin-loading controls and quantified by densitometric scanning (anti-EGFR = black; anti-pCrk251 = blue; anti-Crk = green; anti-Abi-Iso2 = red).

We investigated the potential interplay between Crk Y251 phosphorylation, loss of Abi1, and Abl activation, by meta-analyzing genomic datasets obtained from annotated next generation sequencing studies in tumor samples of GBM cancers as described by CBioportal cancer genome analysis [26, 27] and Oncomine database analysis [28]. As shown in Figure 1A, Crk mRNA expression levels are up-regulated in GBM as compared to normal tissue in datasets mined from several independent datasets as collectively summarized from Shai and colleagues [29], TCGA database [30], Murat and colleagues [31] and Bredel and colleagues [32]. By contrast, Abi1 mRNA expression levels are downregulated in datasets using from TCGA database [30], Sun and colleagues [33] and Lee and colleagues [34] (Figure 1B). Similarly, copy number alteration analysis of datasets from Beroukhim and colleagues [35] and TCGA database (normal = 321, glioblastoma = 472) indicate that Abi1 gene copy number is significantly reduced in GBM compared to normal tissue (Figure 1C). Moreover, these trends of inverse relationship between Crk and Abi1 expression correlated with survival outcomes when accessing the gene expression profiles using SurvExpress analysis [36]. Taken together, these results support the idea that high Crk (Figure 1D) and low Abi1 (Figure 1E) gene expression correlates with poor patient survival.

Reciprocality in Crk and Abi1 expression noted above led us to survey Abi1 expression levels in several GBM cell lines that include U118MG, U138MG, A172, U87MG, T98G and HS683 (Figure 1F) as well as patient-derived GBM samples. Using an Abi1-Iso2 specific antibody 4E2 (Abcam), 3 of the 6 lines, namely T98G, HS683 and U87, had lower or negligible levels of Abi1-Iso2 as compared to SVGP12 cells, an immortalized line derived from normal human astrocytes. To translate these observations to explore the clinicopathological significance of EGFR, Crk, Crk pY251 and Abi1 protein expression in GBM, we performed Western blot analysis of patient GBM tissue samples (*n* = 32) *versus* matched normal tissue samples (*n* = 18), and consistent with the data using cell lines, GBM samples have up-regulated protein levels of EGFR (1.7 fold), CrkpY251 (1.5 fold), Crk (1.45 fold) and decreased level of Abi1-Iso2 (0.82 fold) (Figure 1G and 1H).

**Figure 2:**
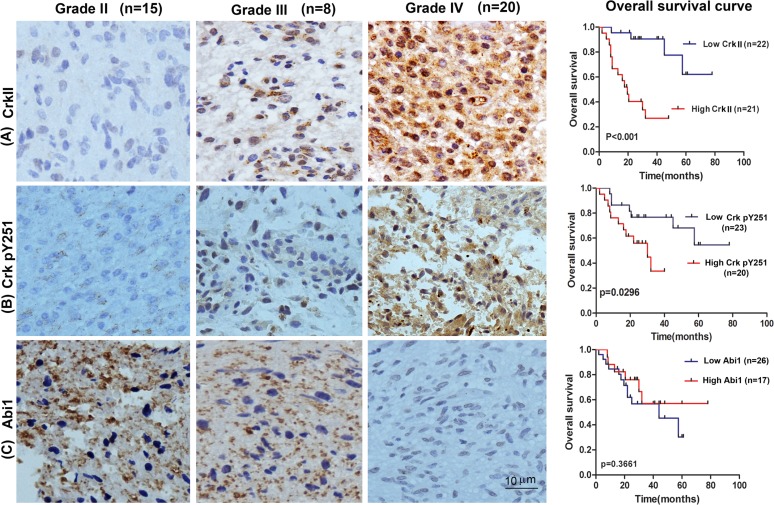
Tissue microarray of GBM patient tumor samples reveals reciprocal expression of Crk and Abi1 in glioblastoma Representative images of upregulated tissue expression of **A**. Crk and **B**. Crk pY251 in Grade IV glioblastoma (middle panels) *versus* Grade I glioma (left panels). Kaplan-Meier survival curves show high expression of Crk and Crk phospho-Y251 are correlated with low overall survival in GBM patients (A-B, right panels). **C**. Abi1 tissue expression is downregulated in Grade IV glioblastoma *versus* Grade I glioma and is correlated with lower overall survival. See also Supplementary Figure S1, S2 and Table 1–3 and Supplementary Tables 1-2.

We next investigated the association of EGFR, Crk, Crk pY251 and Abi1-Iso2 protein expression in the tumor tissues with clinical and pathologic characteristics of glioma patients as previously indicated [37]. We performed immunohistochemical staining (IHC) in TMA containing 43 archived paraffin-embedded glioma cancer samples (Figure 2) and found that Crk and Crk pY251 expression were upregulated in un-differentiated (G4) GBM cancer tissues as compared to lower grade G2 and G3 glioma cancer tissues (Figure 2A-2B, Table 1 and 2. *p* = 0.02, *p* = 0.029, respectively). Inversely, Abi1-Iso2 expression was downregulated in undifferentiated (G4) GBM cancer tissues as compared to lower grade G2 and G3 glioma cancer tissues (Figure 2C and Supplementary Table 2). Moreover, a significant clinicopathological correlation between EGFR expression and phospho Crk Y251 expression in G3-G4 GBM samples (Table 3. *P* = 0.033) was noted by chi-square test and that Crk and EGFR expression were significantly associated with the age of glioma patients (Table 1 and Supplementary Table 1. *p* < 0.001 and *p* = 0.048). No significant relationship was found between EGFR, Crk, Crk pY251 and Abi1 protein expression with the gender of glioma patients (Tables 1–2, and Supplementary Tables 1-2).

**Table 1:** Association between CrkII expression and clinicopathological factors of glioma patients

Variables	CrkII protein expression (n=43)		*P* value
	High (n=21)	Low (n=22)	
**Age**			**<0.001**
≤47	5	17	
>47	16	5	
**Gender**			0.887
Male	10	10	
Female	11	12	
**Grade**			**0.02**
G1-2	3	13	
G3-4	18	9	

**Table 2:** Association between Phospho-Crk Y251 expression and clinicopathological factors of glioma patients

Variables	Phospho Crk Y251 protein expression (n=43)		*P* value
	High (n=20)	Low (n=23)	
**Age**			0.172
≤47	8	14	
>47	12	9	
**Gender**			0.298
Male	11	9	
Female	9	14	
**Grade**			0.029*
G1-2	4	12	
G3-4	16	11	

**Table 3:** Association between EGFR and Phospho-Crk Y251 expression and clinicopathological factors of glioma patients

Variables	EGFR/p-Crk Y251 protein expression (n=43)				*P* value
	+/+(n=13)	+/−(n=7)	−/+(n=7)	−/−(n=16)	
**Age**					0.103
≤47	3	4	5	10	
>47	10	3	2	6	
**Gender**					
Male	8	2	4	6	0.403
Female	5	5	3	10	
**Grade**					
G1-2	1	2	4	9	**0.033**
G3-4	12	5	3	7	

The prognostic value of Crk, Crk phosphoY251 and Abi1 for overall survival in glioma cancer patients was evaluated by comparing the patients with high and low Crk, Crk pY251 and Abi1 expression. According to Kaplan-Meier survival analysis, patients with high Crk and Crk phosphoY251 expression had distinctly lower overall survival rates than those with low Crk and Crk phosphoY251 expression (Figure 2A-2B right panels, *p* < 0.001 and *p* = 0.0296 respectively). By contrast, although low expression of Abi1-Iso2 appeared to have lower overall survival, this could not reach statistical significance (Figure 2C right panel, *p* = 0.366). H&E staining were performed on all the specimens to assess the tumor grades (Supplementary Figure S1).

Based on the range of expression of *ABI1* and *CRK* in human GBM samples, we hypothesized that the low Abi1/high Crk signatures observed in a subset of human GBM may represent a biologically distinct subset that favors a more aggressive phenotype, we selected cases with high levels of *CRK* and low levels of *ABI1* based on RNA-Seq data deposited into TCGA, and compared their gene expression to cases with intermediate levels of *CRK* and *ABI1*, which would identify altered phenotypes when Crk and Abi1 interaction is dysregulated or uncoupled (Supplementary Figure S2A). Notably, Gene Set Enrichment Analysis revealed that pathways involved in regulating cell invasion, including E-cadherin signaling (*P* = 0.02), were enriched when Crk and Abi1 expression was altered (Supplementary Figure S2B and S2C).

### Crk Y251 phosphorylation is a common feature in GBM cell lines to promotes Abl transactivation

**Figure 3:**
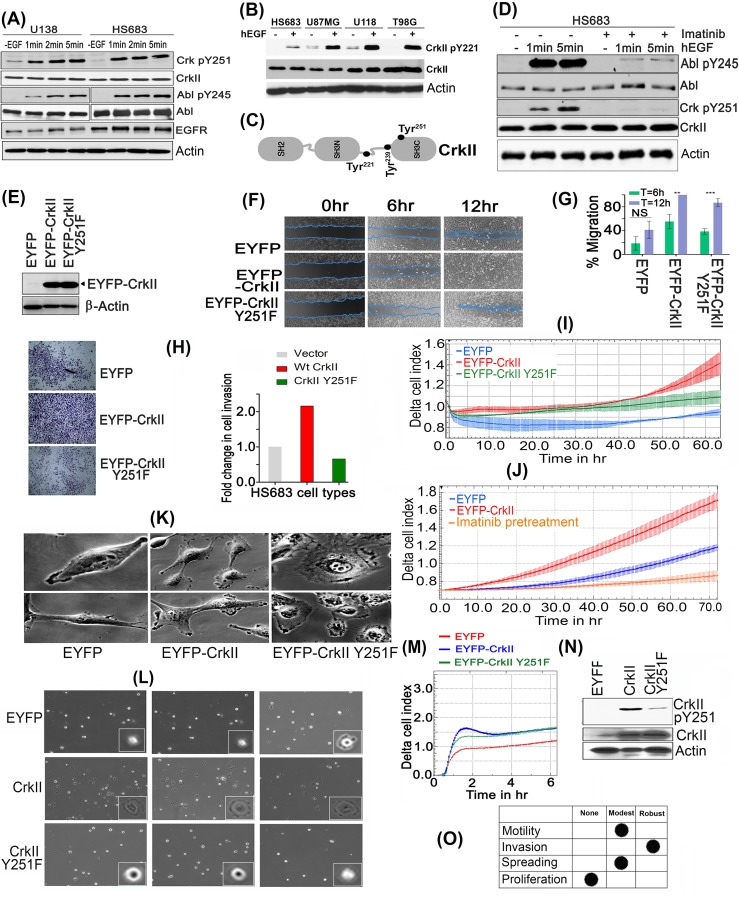
CrkY251 phosphorylation drives Abl transactivation and regulates distinct biological phenotypes in GBM cell lines **A.** Upon stimulation with hEGF, Crk and Abl kinase are phosphorylated within 1min in U138 and HS683 GBM cells. **B.** Canonical Crk signaling is seen by CrkY221 phosphorylation by hEGF in GBM cell lines. **C.** Schematic figure of Crk indicating Tyr 221, 239 and 251 in context of its modular domains. **D.** Parental HS683 cells were pretreated with or without 10uM Imatinib and stimulated with or without hEGF after which detergent lysates were made and immunoblotted with Abl pY245 and Crk pY251 antibodies. **E.** Western blot analysis performed to assess the stable expression of MSCV-EYFP (Empty vector), MSCV-EYFP-Crk and MSCV-EYFP-CrkY251F in HS683 cells. **F.** Wound healing assay: Cell migration of the HS683 stable cells was assessed by imaging the rate of wound healing by serum starved, hEGF stimulated cells in triplicate samples at 6 and 12 hours time points. **G.** Percentage wound healing was calculated in all cell lines and data were represented as mean ± SEM (*n* = 3) (*P* < 0.05). **H.** Cell invasion by Matrigel-coated Boyden chambers: Post-serum starvations for 12-16hrs, 10,000 cells were seeded in serum free media. 10% FBS containing media was used as chemoattractant in the lower chamber. Cell were fixed, stained and imaged as described (see methods) and quantified to represent data in fold change in cell invasion (left panel). **I.** Cell invasion using real-time xCELLigence-based assay: Overnight serum starved cells were seeded in serum free media in triplicates in 40000 cells/well. Cell invasion was assayed every 15min for indicated time using 10% FBS containing media as chemoattractant (*P* < 0.05). **J.** xCELLigence assay to test the effect of Imatinib treatment on cell invasion of Hs683 cells. **K.** Distinct morphological features by cells expressing vector (EYFP), Crk, or CrkY251F mutant. **L.** Micrographs of cell spreading on fibronectin coated dishes: 1000 respective cells were seeded on fibronectin pre-coated dishes (10ug/ml) and images were taken from multiple fields (3 fields/cell line shown) 30min later using 40X objective of phase contrast microscope. **M.** Real-time xCELLigence-based cell spreading assay on fibronectin-coated CIM-16 plates seeded with 10,000 vector, Crk, CrkY251F expressing HS683 cells using 10%FBS as chemoattractant. **N.** CrkY251 phosphorylation by fibronectin in stable cell lines: The stable cell lines that were seeded on fibronectin dishes for 30min were harvested, lysed and immunoblotted for endogenous and EYFP- CrkY251 phosphorylation status. **O.** Tabular summary of the CrkY251-mediated biological phenotypes observed. See also Supplementary Figure S3, S4 and S5.

To translate the Crk, Crk pY251 and Abi1-Iso2 expression datasets into a mechanistic outcome, we investigated how these adaptor proteins impinged on Abl activation in a genetically amenable system. Previously, we reported that EGF-inducible phosphorylation of Crk on Tyr251 resulted in the transactivation of Abl kinase, and affirmatively regulated cell motility in MDA-MB-468 breast cancer cell lines [13, 38]. Since amplification of EGFR is an important driver of tumorigenesis in GBM (as evident from cancer genome sequencing analysis) [30, 39], we explored the Crk Tyr251/Abl axis in several GBM cells lines, that de-regulate EGFR, using phosphospecific antibodies to AblY245 and CrkY251 (Supplementary Figure S3). As shown in Figure 3A, Supplementary Figure S4A and S4B, both CrkY251 and AblY245 were immediately phosphorylated after 1min of EGF stimulation in multiple GBM lines that include U138, HS168, T98G and U118MG, suggesting CrkY251 phosphorylation is a routine post-receptor event following EGF stimulation in EGFR-expressing GBM. Simultaneously to CrkY251 phosphorylation, endogenous CrkY221 phosphorylation was also observed in all GBM cells lines treated with EGF (Figure 3B, 3C).

To investigate whether phosphorylation on Crk Y251 in GBM cell lines was mediated directly by EGFR, or indirectly by Abl, HS683 cell lines were pretreated with Imatinib prior to EGF administration (Figure 3D). As shown, Imatinib pretreated cells showed a marked decrease in CrkY251 phosphorylation, which correlated with the decreased levels of Abl activation (pY245 phosphorylation). Similar results were observed when EYFP-Crk was stably (ectopically) expressed in HS683 cell lines, whereby treatment with EGF resulted in enhanced CrkY251 phosphorylation and Abl kinase transactivation (Supplementary Figure S4C and Figure S4D). This suggests that in GBM cells, CrkY251 phosphorylation is regulated mainly by Abl, with a minor, but finite, component contributed by EGFR.

### Crk Y251 phosphorylation controls aggressive cancer phenotypes in GBM cells

To explore the relationship between CrkY251 phosphorylation and aggressive cancer phenotypic responses in GBM, we created stable lines that express EYFP, EYFP-Crk or EYFP-Crk Y251F. After selection, cells were subjected to flow- cytometer-based sorting for comparable expression by geometric mean intensity (Figure 3E), after which wound healing/ scratch assays were performed. As shown in Figure 3F-3G, HS683 cells expressing WtCrk showed enhancement in wound healing, while Crk Y251F overexpressing cells had reduced rate of cell migration at both time points, although notably the motility of Crk Y251F-expressing cells was still significantly higher than control EGFP-expressing cells.

Replicate experiments were performed to investigate effects of CrkY251 phosphorylation on EGF-mediated cell invasion, using Matrigel pre-coated Boyden chambers and xCELLigence-based real-time invasion assays on the aforementioned cell lines described above (Figure 3H, 3I). WtCrk HS683 cells strongly increased cell invasion through a Matrigel membrane, while Y251F mutation virtually abrogated this effect. Furthermore, pretreatment with Imatinib significantly reduces invasion of HS683 GBM cells indicating a role for Abl kinase activity in regulating cell invasion of these cells (Figure 3J). Since Abl activation has been shown to positively regulate invasion [40-42], and CrkY251 phosphorylation induces Abl trans-activation, these results suggest that the robust suppression in cell invasion by CrkY251F expressing HS683 cells are likely due to suppressed endogenous Abl activity. Consistent with these phenotypic responses, Crk expressing HS683 cells have more abundant and pronounced lamellipodia (Figure 3K), while CrkY251F cells had a more flattened morphology that showed an early decrease in cell spreading on pre-coated fibronectin dishes (Figure 3L) and using real-time cell spreading using xCELLigence RTCA DP (Figure 3M, 3N). Cell proliferation rates between vector, WtCrk and Crk Y251F expressing stable HS683 cell lines were not significantly different (Supplementary Figure S5).

### Abi1 competes with Crk in binding the C-terminal Proline rich domain (PRD) of Abl to suppress Abl activity

**Figure 4:**
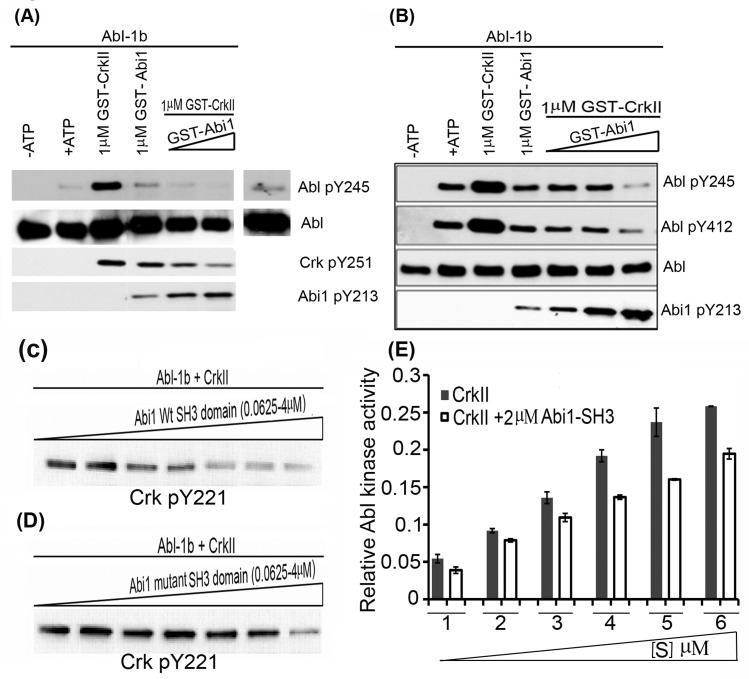
Abi1-Iso2 competes with Crk for Abl kinase Proline-rich domain *In vitro* kinase assay performed with bacterially purified WtCrk and Abi1-Iso2 and Abl-1b purified from SF9 cells. 10nM Abl-1b was incubated with 1uM Crk alone or in presence of increase concentrations of Abi1-Iso2 with ATP at 25°C for 10mins. **A.** Crk mediated Abl transactivation was significantly suppressed in samples containing Abi1-Iso2 as shown by Abl pY245 blots. **B.** In another variation, Abi1 alone downregulates Abl activation alone and in presence of Crk in dose- dependent manner as indicated by Abl pY412 blot. **C.**-**D.** *In vitro* kinase assay to assess Abi1 SH3 domain on Abl kinase activity: Abl-1b and Crk were co-incubated with either **C.** Wt SH3 domain or **D.** mutant SH3 domain of Abi1-Iso2 in increasing amounts. In a dose-dependent manner Abi1 Wt-SH3 domain significantly suppresses Abl-1b-mediated CrkY221 phosphorylation (C), whereas mutant SH3 domain looses the capacity to suppress aforementioned CrkY221 phosphorylation (D). **E.** Competitive kinase assay: Abl kinase was co-incubated either with Crk alone or Crk and 2μM Abi1-Iso2 SH3 domain in kinase buffer. After 30min, Abl kinase activity was assessed by CrkY221 phosphorylation. Data are represented as mean ± SEM and *n* = 3 (*P* < 0.05).

To directly test whether Abi1 competes for Crk-inducible Abl activation *in vitro*, we performed competitive *in vitro* kinase assay using bacterially expressed and purified GST-Crk and GST-Abi-Iso2 and baculovirus-produced non-GST tagged Abl 1b purified in the presence of 10μM Imatinib to prevent artificial activation (Figure 4A). GST-Crk transactivated Abl kinase, whereas GST-Abi1 failed to activate Abl kinase under these conditions (lane 2 *versus* lane 4). In fact, when co-incubated with GST-Crk, GST-Abi1 reduced the Crk-mediated Abl transactivation in a concentration dependent manner to background levels (Figure 4A-4B). Concomitantly, Abl dependent CrkY251 phosphorylation was also reduced upon Abi1 titration (Figure 4A) while Abi1 Y213 phosphorylation (indicative of Abl and Abi1 binding) was increased (Figure 4A-4B) in a manner that positively correlates with the Abi1-Iso2 concentration.

Further, to assess the role of SH3 domain of Abi1-Iso2 in a competition assay, Wt or mutant SH3 domains of Abi1-Iso2 were bacterially expressed, purified and co-incubated in kinase buffer with Abl-1b and WtCrk in different molar ratios. Consistently, we found that with increasing molar ratio of WtSH3 domain of Abi1, CrkY221 phosphorylation was significantly reduced in an *in vitro* kinase assay (Figure 4C), while suppression of CrkY221 phosphorylation by mutant Abi1-Iso2 SH3 domain was much less apparent (compare Panel C and D in Figure 4). Finally, we performed competitive kinase assay using bacterially purified Crk and Abi1- Iso2 SH3 domain. We found that upon co-incubation of Abi1-Iso2 SH3 domain, the enzymatic reaction velocity of Abl kinase (that is transactivated by Crk) is significantly reduced at all substrate concentrations (Figure 4E), further indicating that the SH3 domains of Abi1-Iso2 and Crk functionally compete.

### Reciprocal role of Abi1-Iso2 in Crk-mediated Abl transactivation and in Crk-mediated cell motility, invasion, and spreading

**Figure 5:**
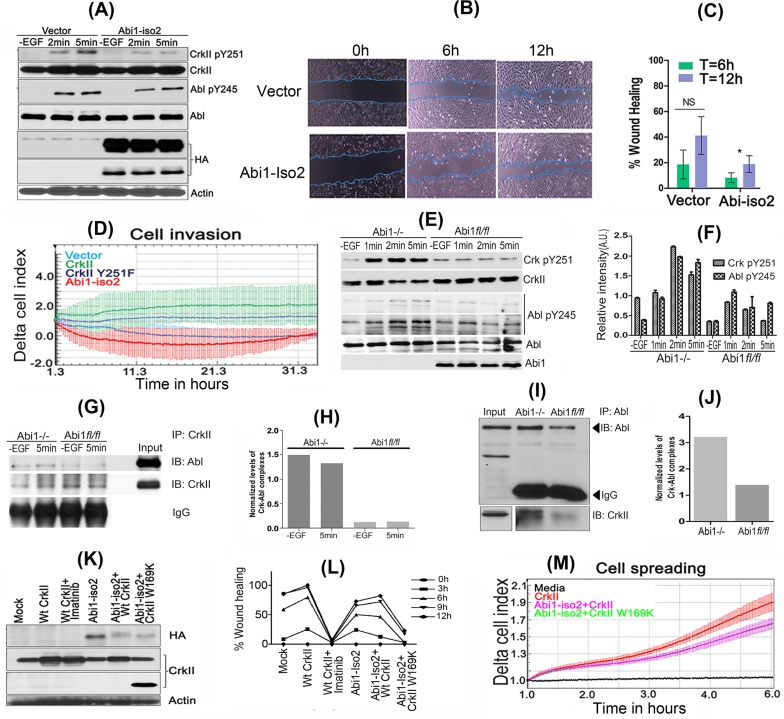
Reciprocal role of Abi1-Iso2 in Crk-mediated Abl transactivation and in Crk-Abl1b axis-mediated cell motility and invasion **A.** HS683 stable cell line overexpressing Abi1-Iso2 shows significant reduction in Crk Y251 and Abl Y245 phosphorylation in time dependent manner. **B.** Wound-healing assay: Cell migration of HS683 stable cells was assessed by imaging the rate of wound healing by serum starved, hEGF stimulated cells in triplicate at 6 and 12hrs time-points. **C.** Percentage wound healing was calculated and represented in the bar graph (*n* = 3) (*P* < 0.05). **D.** Real-time xCELLigence assay for cell invasion: Cells were grown to 80% confluency, serum starved and seeded in triplicates in matrigel coated upper chambers of CIM plates. 10% FBS containing media was used as chemoattractant in the lower chamber of CIM plate. Change in Cell index was measured every 15min for 36-48 hours. **E.** Abi1−/− and Abi1 *fl/fl* MEFs were serum-starved overnight and stimulated with 100ng/ml of hEGF. Enhanced CrkY251 and AblY245 phosphorylation was observed in Abi1−/− MEFs as compared with Abi1 *fl/fl* cells. **F.** Densitometry of phosphoprotein bands were calculated, normalized by ImageJ and represented in bar graphs. **G.**-**H.** Crk and Abl kinase complexes that form with or without EGF treatment in Abi1−/− MEFs were investigated by immunoprecipitation of Crk after diluent or hEGF stimulation. **I.**-**J.** Reverse immunoprecipitation by Abl antibody and immunoblotting with Crk antibody was performed. **K.**-**L.** Wound healing/scratch assay: Abi1−/− MEFs, were transiently transfected as indicated. 48hrs post-transfection, cells were serum starved for 16hrs and assayed for wound healing as described (see methods). **M.** Microimpedance-based cell spreading assay: 10,000 cells transfected as described in panel K were seeded on fibronectin coated E-plate 16 and assessed by real-time cell spreading assay every minute by xCELLigence RTCA DP. Data are represented as mean ± SEM, and *n* = 3 for Panel C and F. See also Supplementary Figure S6.

To more formally establish a causal role for Abi1-Iso2 that functionally impinges on Crk Y251 phosphorylation and Abl activation in GBM cell lines, we stably expressed Abi1-Iso2 in HS683 cells by retroviral transduction. After serum starvation and stimulation with hEGF for the indicated time points, HS683 cells stably expressing Abi1-Iso2 showed markedly lower CrkY251 and AblY245 phosphorylation as compared to vector expressing cells, suggesting a mode of competition between Abi1 and Crk that reciprocally and functionally regulates Abl kinase (Figure 5A). Ectopic expression of Abi1 in HS683 cells also reduced cell migration rate (Figure 5B-5C), and significantly abrogated cellular invasive properties (Figure 5D).

We also analyzed the reciprocal effects of Abi1 and Crk on Abl signaling in Abi1(−/−) MEFs reconstituted with Abi1 to rule out the possibility that other Abi1 isoforms may compensate for Abi1-Iso2 in the HS683 tumor cells. Consistent with the aforementioned *in vitro* studies and reconstituted HS683 cells, Abi1 (−/−) cells stimulated with EGF showed enhanced CrkY251 phosphorylation and AblY245 phosphorylation (Figure 5E, 5F). Moreover, Abi1 (−/−) cells had higher steady state and EGF-inducible complex formation between Abl and Crk in co-immunoprecipitation studies (Figure 5G-5J). Finally, when we transiently transfected Abi1 (−/−) MEFs with WtCrk, Abi1-Iso2 or co-transfected Abi1-Iso2 with Wt or SH3 mutant Crk and performed scratch assay, (Figure 5K-5L), the rate of wound healing was highest in cells expressing WtCrk and Abi1 expression delayed the rate of wound healing. Likewise, using fibronectin-coated E-plates and real-time cell spreading assay, we found that Abi1-Iso2 suppresses Crk mediated cell spreading of Abi1−/− MEFs upon co-transfection (Figure 5M) while the Crk SH3N mutant CrkW169K cotransfected with Abi1 iso-2 also showed reduced cell spreading (See also Supplementary Figure S6).

### Abi1-Iso2 binds Abl kinase in partially open conformation to suppress its activation

**Figure 6:**
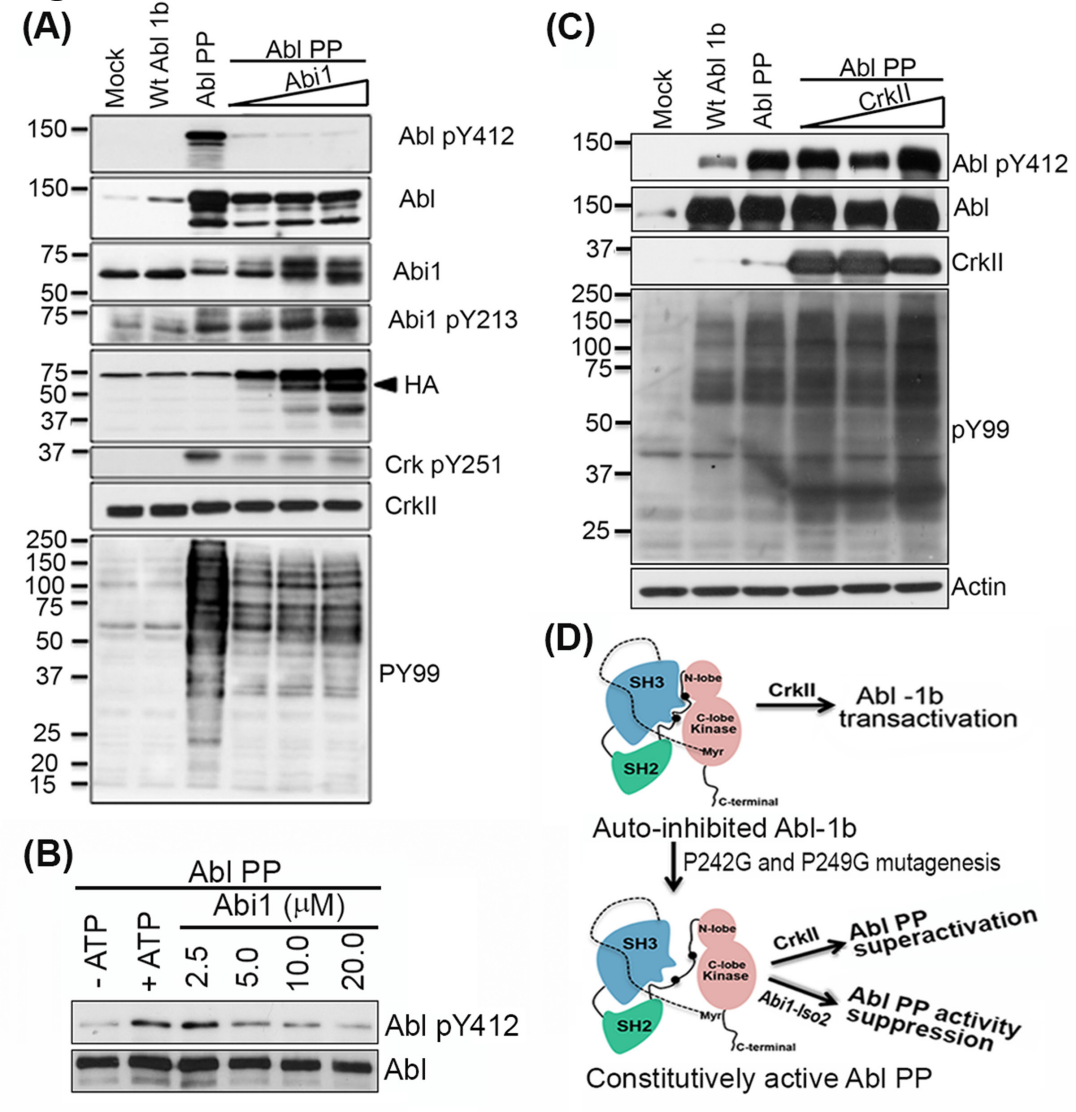
Abi1-Iso2 can bind to open conformation of Abl kinase to suppress its activity *in vivo* **A.** HEK293T cells were transfected with plasmids encoding WtAbl-1b or constitutively active, partially open conformation Abl PP alone or co-transfected with increasing amount of Abi1-Iso2 plasmid DNA. Abl PP constitutive activity is downregulated to markedly low levels (Abl pY412 blot) that correlates with Abi1-Iso2 expression level (HA blot) in cells. Abi1 Y213 phosphorylation increases with increasing amount of Abi1 in these cells. Endogenous Crk Y251 phosphorylation and general phosphotyrosine levels (PY99) that are mediated by Abl PP are downregulated by Abi1-Iso2. **B.** Crk co-transfected with Abl PP in the similar experimental design superactivates Abl PP as indicated by PY99 blot. **C.** *In vitro* kinase assay using Abl PP and Abi1-Iso2: Abl PP was co-incubated with Abi1-Iso2 in kinase buffer for 30min, reactions were boiled and immunoblotted for Abl pY412. **D.** Schematic representation of effect of Crk and Abi1-Iso2 on differential regulation of Abl PP activity.

To better evaluate the biochemical mechanisms that underlie the opposing effects of Crk and Abi1-Iso2 on Abl activity, we tested whether Crk and Abi1-Iso2 can impinge on Abl when Abl is expressed in a partially open and active configuration [24, 38]. We HEK293T cells were co-transfected with increasing DNA concentrations of Abi1-Iso2 and the Abl PP variant (previously described by [43] that has P242G and P249G mutations in the SH2- kinase domain linker that render it a partially open conformation and constitutively active) (Figure 6A). Under these conditions, Abi1-Iso2 completely blocked the constitutive activity of Abl PP as indicated by Abl Y412 phosphorylation in the activation loop, as well as the substrate-level phosphorylation observed with a PY99 blot. Abi1-Iso2 co-transfection also concurrently induced Abi1 Y213 phosphorylation (indicative of Abi1-Iso2 and Abl binding) and decreased endogenous CrkY251 phosphorylation mediated by Abl PP, affirming the competitive role of Abi1 in the Crk-Abl complex. Furthermore, we tested such a negative regulation of Abl PP constitutive kinase activity by Abi1-Iso2 using immunoprecipitated Abl PP from HEK293T cells as previously described [38] and bacterially Abi1-Iso2 proteins in an *in vitro* kinase assay. Consistent with the co-transfection experiment, co-incubation of Abl PP with purified Abi1-Iso2, significantly suppresses Abl PP's constitutive kinase activity (shown by reduction in Abl pY412 phosphorylation) in a concentration-dependent manner (Figure 6B). In contrast, when similar experiment was performed with WtCrk, notably, Crk induced a further increase in Abl PP Y412 phosphorylation and consequent increase in PY99 levels, suggesting superactivation by Crk (Figure 6C). These data suggest that Crk Y251 phosphorylation downstream of Abl, potentiated by loss of Abi1 expression, cause a feed forward mechanism leading to hyperactivation of Abl (Figure 6D).

## DISCUSSION

Here we demonstrate an important mechanism whereby the Abl interacting adaptor proteins Abi1 and Crk differentially impinge on Abl to regulate invasive GBM. Using several cell lines as a model of classical GBM, a type characterized by high endogenous levels of EGFR, we found that CrkY251 phosphorylation, induced downstream of EGFR activation, is a frequent event in GBM cells and correlates with aggressive cancer phenotypes. Tyrosine phosphorylation of Crk Tyr251, in turn, results in the trans-activation of Abl kinase, mediated at least in part, by direct binding between Crk and Abl kinase and pTyr251-mediated displacement of the auto-inhibited structure of Abl [38]. We also show that loss of Abi1, another SH3-domain containing adaptor protein that interacts with the PRD of Abl, competes for Crk binding, and antagonizes the effect of Crk on Abl-dependent motility and invasion in GBM cell lines. Together, these data suggest that Abi1 and Crk impart a reciprocal regulation on Abl kinase (Crk positively regulates while Abi1 negatively regulates), and that loss of the Abi1 gene in GBM functions in part by enhancing the Crk Tyr251 Abl axis to promote aggressive behavior in tumor cells.

**Figure 7:**
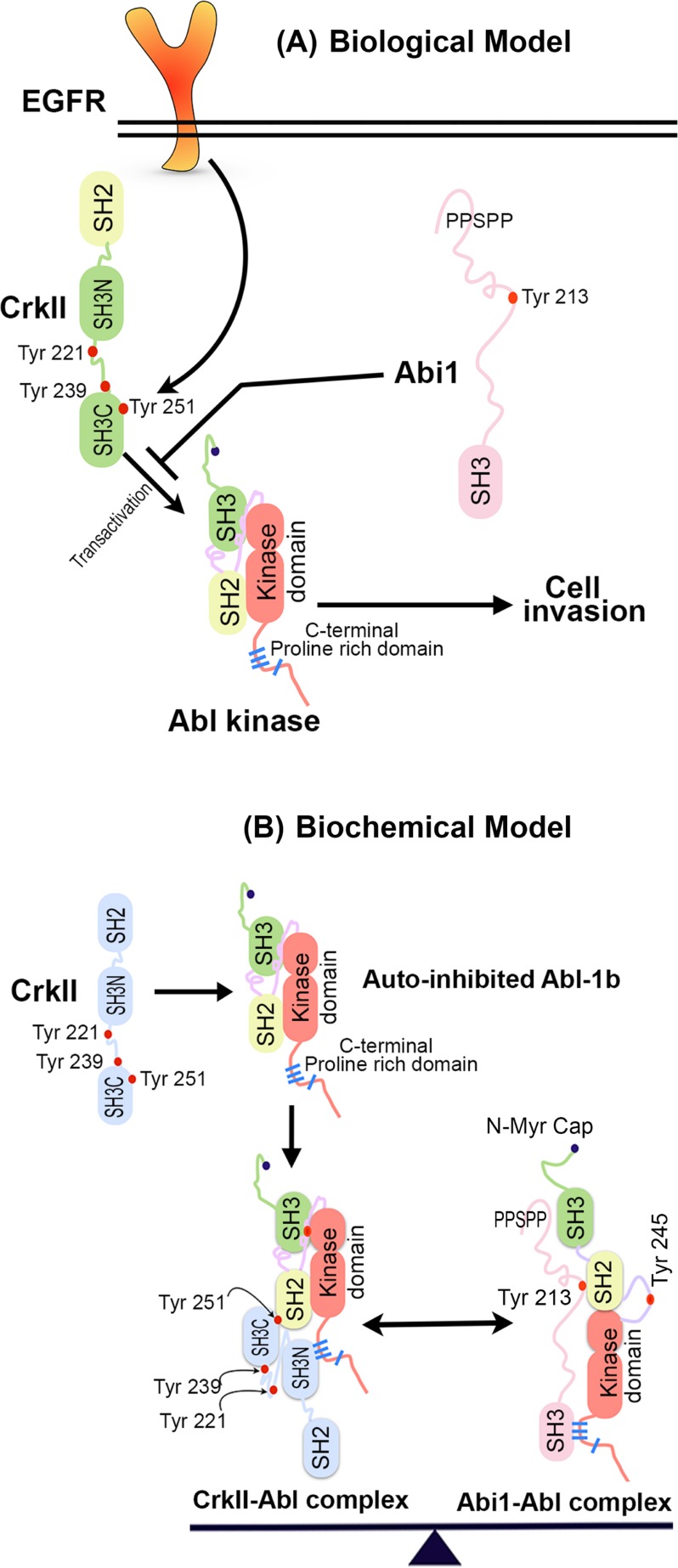
**A.** Biological model for role of Abi1-Iso2 in regulation of CrkY251 mediated Abl transactivation and cell invasion: Downstream of EGFR, which is amplified in classical GBM, CrkY251 becomes phosphorylated and drives Abl kinase transactivation that results in cell invasion. Loss of Abi1 and gain in Crk expression in GBM results in enhanced invasion via an amplified Crk-Abl transactivation pathway. **B.** Crk and Abi1 interact with Abl kinase with different itineraries: Proposed model by which Crk SH3N binds to the C-terminal PRD of Abl and leads to phospho Tyr 251 mediated Abl transactivation. In this partially open conformation model of Abl kinase, SH3 domain of Abi1-Iso2 competes with SH3N domain of Crk for binding to C-terminal proline-rich domain of Abl kinase. The Abi1-Abl kinase complex is further stabilized by interaction between N-terminal proline rich sequences of Abi1 and SH3 domain of Abl kinase and Abi1 pY213 and Abl SH2 domain. In homeostasis, relative stoichiometry of Crk-Abl kinase complexes and Abi1-Abl kinase complexes are regulated that creates a binary molecular switch mechanism, thus creating a fine-tuning system of Abl kinase activity.

While cells expressing Crk Y251F showed defects in several cytoskeletal pathways that include spreading on fibronectin, decreased motility, and decreased invasion through a matrigel plug, the effects on the latter were robust and sustainable (Figure 3O). For example, the Crk Y251F-mediated effects on adhesion/spreading and motility were relatively modest, where defects were generally delayed in response and mutant-expressing cells were eventually functional in these capacities. On the other hand, Crk Y251F expressing GBM cells exhibited a sustained and durable suppression in invasion through matrigel, an event confirmed using real-time microimpedance-based xCELLigence technology. These data suggest that the Crk-induced cell motility and Crk-induced cell invasion may be governed by distinct pathways. Indeed, our studies are consistent with previous finding that Crk-inducible motility is primarily regulated by the canonical SH2 and SH3 domain pathway that links tyrosine phosphorylated proteins to DOCK180 and Rac1 activation. In contrast, the present studies suggest that the non-canonical pathway, mediated by the SH3N->pSH3C domains and Abl activation, may be the preferential pathway that regulates invasion (Figure 7).

An important consequence of the aforementioned scenario, in which Crk and Abi1 compete for the same proline-rich motifs in Abl, implicate a net equilibrium in these adaptor proteins exist that can shift the equilibrium towards Abl activation or Abl inactivation depending on the net balance of Crk and Abi1 expression. Indeed, using meta-analysis of genomic datasets derived from human GBM, a general trend is observed towards Crk up-regulation and Abi1 down-regulation. Moreover, using a combination of *in vitro* studies with recombinant proteins, Abi1(−/−) and Crk(−/−) MEFs, and genetically modified GBM cells, our studies show that Abi1 functionally suppresses Crk binding to Abl, functionally suppresses Crk Tyr251 phosphorylation, and functionally suppresses Crk-mediated enhancement in cell motility and invasion. Together, these data are consistent with a potential tumor suppressor role for Abi1 in GBM, and raise a paradigm by which the non-canonical Crk pY251 pathway becomes functionally operational in tumor cells that down-regulate Abi1.

Indeed, both experimental results and the accompanying database mining analysis for Crk and Abi1 suggest that Crk Tyr251 phosphorylation might be informative as a biomarker for invasive GBM. The expression studies shown here support the idea that high levels of Crk, pCrkY251, and EGFR predict aggressive behavior of GBM and patient survival outcomes. Moreover, the fact that Crk Tyr251 can also be phosphorylated by EGFR [38] indicates that the Tyr251 Crk/Abl axis might be particularly relevant in classical GBM, as EGFR gene amplification and overexpression is a particularly striking feature of this tumor type, which is among the most aggressive and invasive forms of this cancer. Clinical studies have investigated the efficacy of Abl kinase inhibitor Imatinib in monotherapy or combination therapy with hydroxyurea [44-46] with little success. We posit that since Crk phosphoY251 may serve as a biomarker for aggressive GBM, in the subset of classical GBM patients that show a high level of CrkY251 phosphorylation and low level of Abi1, Imatinib treatment may prove beneficial to reduce invasive properties of tumor and improve overall survival.

In summary, our studies have identified a novel regulatory network by which Abi1 and Crk reciprocally interact with Abl kinase to drive the malignant phenotypes in EGFR-expressing human GBM cells (Figure 7A, 7B) and functionally that the Crk non-canonical pathway is unleashed by the loss of Abi1.

## MATERIALS AND METHODS

### Patients and specimens

GBM tissues were histopathologically diagnosed at First Affiliated Hospital of Wenzhou Medical University from 2013 to 2015. Normal brain tissue samples were obtained during autopsy, after accidental death, with no clinical or pathological evidence of brain tumor or other abnormality. All GBM and normal tissues were immediately shock frozen in liquid nitrogen and stored at −80°C. The pathological grade of GBM was defined according to WHO-criteria. This study was approved by the Board and Ethical Committee of Wenzhou Medical University. Written informed consents were obtained from all patients who enrolled in this study.

### Tissue microarrays (TMA) and immunohistochemistry (IHC)

Glioma TMA containing a total of 43 formalin-fixed, paraffin-embedded tissue sections (4μm thickness) were constructed as previously described [37]. IHC was carried out according to previously described method [37]. The primary antibodies used were CrkII (1:150, sc-9004), Crk Tyr251 (1:100, ECM Biosciences), EGFR (1: 100, sc-373746) and Abi1 (1:70, ab72318). The IHC staining was reviewed and scored independently by two pathologists who were blinded to the clinicopathological features of the specimens on the TMA. For immunohistochemical quantification, percentage of positive cells and staining intensities were evaluated. The percentage of positive cells was scored from 1-4 and the intensities of staining were graded from 0-3. The two scores were multiplied to generate an immune-reactive score (IRS) ranging from 0-12. Protein expressions were classified into 2 categories according to IRS score: “Low” level scores ≤ 6) and “High” level (Scores >6).

### Western blot analysis and immunoprecipitation assays

Cells were lysed in 1% HNTG buffer supplemented with sodium vanadate, sodium molybdate and protease inhibitor cocktail. Western blot analyses were performed using EGFR, phospho-Y221 CrkII, previously described phospho- Crk Y251 (CST), anti-pY245 Abl, and anti-pY412 Abl (Cell Signaling Technology Inc.), phosphotyrosine antibody PY99, GST (Santa Cruz Biotechnology Inc.), Abl Ab-3 (Calbiochem), and Abi1 1B9 antibodies. For immunoprecipitation, cell lysates were made in 1% HNTG buffer, incubated with respective antibodies and supplemented with protein A/G agarose beads. After incubation and repeated washing with 0.1% HNTG buffer, samples were boiled and analyzed by immunoblotting.

### *In vitro* kinase assays

10nM Abl 1a was incubated with a 100-1000 fold molar excess of GST, GST-CrkII or GST-Abi1 isoform2 proteins in kinase buffer (50mM Tris-Cl, 3mM DTT and 10mM MgCl_2_). The reaction mixtures were kept on ice with intermittent mixing for 1hr. 0.1mM ATP was added to the individual reaction mix and incubated in at 25°C for 10min. The reactions were terminated by addition of SDS sample buffer and analyzed by immunoblotting.

### Real-time cell migration and Invasion assay using xCELLigence

Glioblastoma cell lines stably expressing EYFP, WtAbi1-Iso2, WtCrkII or CrkII Y251F were serum starved overnight. DMEM containing 100ng/ml human EGF was added as chemoattractant in lower chamber of RTCA-DP CIM-16 plate coated with 1μg/ml of fibronectin. Respective cell lines, suspended in DMEM (40000 cells/100uL), were added in triplicates in upper chamber and change in Cell Index (CI) were measured every 5min for mentioned time periods. For invasion assays, an additional 10% matrigel plug was used in the upper chamber.

### Wound-healing assay

Cells were serum starved overnight, washed with 1X PBS and supplemented with 100ng/ml hEGF containing medium. Using a 200uL sterilized tip, single scratches/ well were made in sterile conditions. Images were taken quickly after this step followed by imaging every 3hrs.

### Bioinformatics analysis

Bioinformatics meta-analysis for genomic and gene expression changes were performed on next-generation sequencing data from multiple studies made available through CBioportal for Cancer Genomics and Oncomine databases. Results were represented as on a log2 scale as Box- Whisker plots. P value of less than 0.003 was considered significant. The gene expression data of CrkII and Abi1-Iso2 were used to classify human GBM cases into high and low groups by SurvExpress analysis. Gene Set Enrichment Analysis (GSEA) was performed on the Broad Institute GenePattern Server using 75th-percentile normalized RSEM gene expression values downloaded from the NCI Data Matrix Portal. Cases of coupled or uncoupled *ABI1* and *CRK* expression were selected based on the normalized expression of these genes relative to the remainder of the dataset.

### Statistical analysis

All the experiments were repeated at least 3 or more times. Unless otherwise mentioned, all measurements are shown as mean ± SEM. ANOVA and Student's t-test were performed as mentioned using GraphPad Prism software. The χ2 test was used to analyze the relationship between respective protein expressions and clinicopathological characteristics. Statistical significance was set at *p* < 0.05.

## SUPPLEMENTARY MATERIAL AND METHODS FIGURES AND TABLES


