# Supplementary material for: Reciprocal regulation of Abl kinase by Crk Y251 and Abi1 controls invasive phenotypes in glioblastoma
Source: Oncotarget. 2015 Oct 12;6(35):37792–807. doi: 10.18632/oncotarget.6096 (PMC4741966; doi:10.18632/oncotarget.6096)
Supplement: Supplementary file 1 [file oncotarget-06-37792-s001.pdf]

Reciprocal regulation of Abl kinase by Crk Y251 and Abi1 controls invasive phenotypes in glioblastoma

Supplementary Material

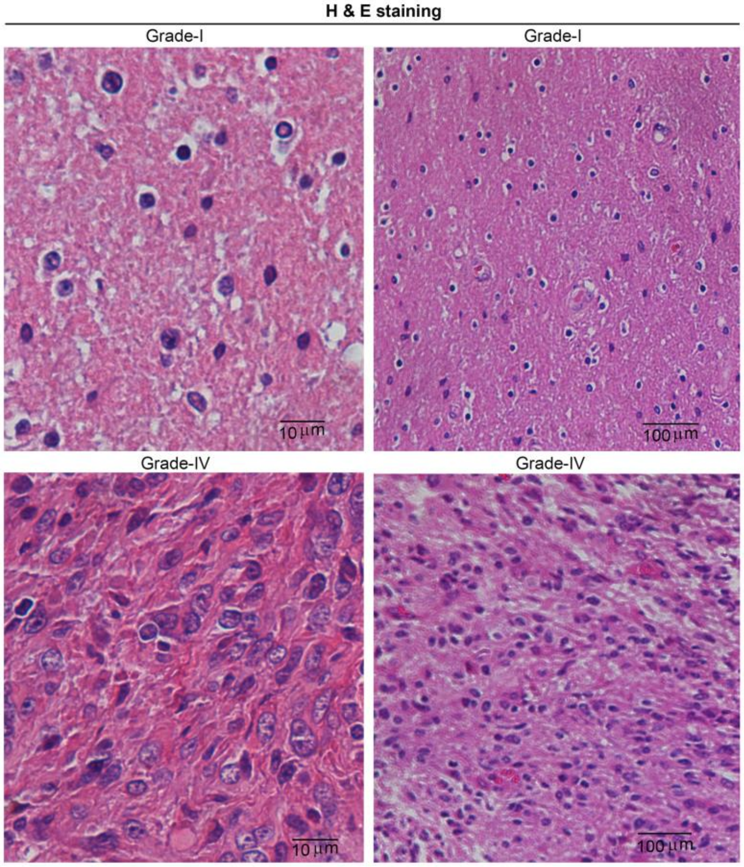

Figure S1: Representative images from H&E staining performed on tissue sections to confirm the grade of specimens with high and low magnifications.

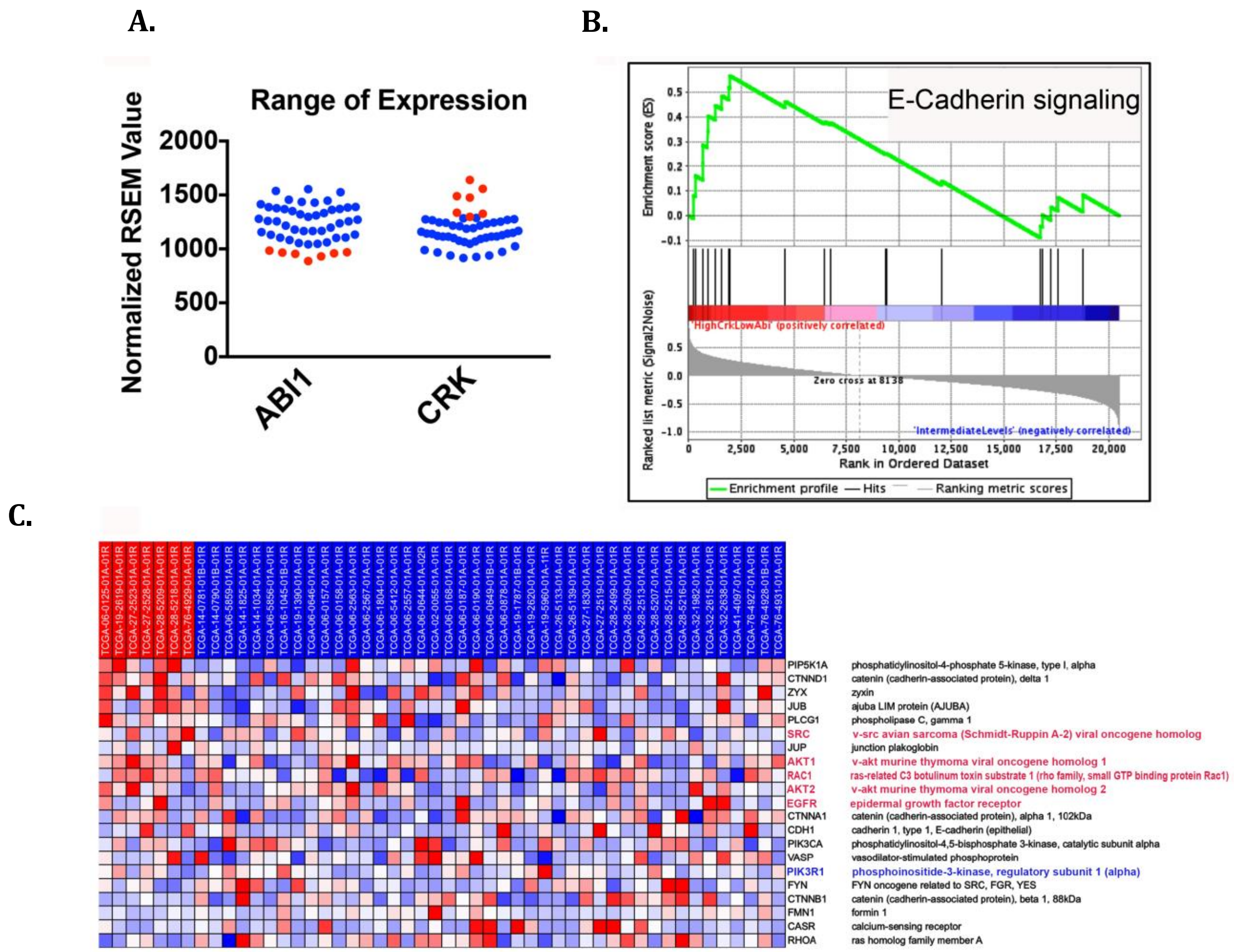

Figure S2: Uncoupled expression of *ABI1* and *CRK* may favor a migratory phenotype. Upper left: A. Normalized RSEM values (of RNA-seq gene expression analysis) for GBM TCGA cases were downloaded from the NCI Data Portal and stratified based on their expression of *ABI1* and *CRK*. Of 528 total cases of GBM in TCGA (and 174 with RNA-seq data), 43 (blue) had both *ABI1* and *CRK* at intermediate levels of expression relative to the sample population (at the 2nd or 3rd quartile), while 7 cases (red) had lower levels of *ABI1* (less than the second quartile) and higher levels of *CRK* (greater than the third quartile). **B.** Gene Set Enrichment Analysis of *ABI*-low/*Crk*-high GBM cases vs. cases with intermediate levels suggested strong enrichment (NES = 1.71) for the "E-cadherin signaling" pathway as indicated in the Schaefer *et al* Pathway Interaction Database. **C:** Examination of the genes involved in the enrichment revealed recurrent up-regulation of growth and motility regulating genes *AKT1*, *AKT2*, *RAC1*, *SRC*, and *EGFR* in the *ABI*-low/*Crk*-high subset, and down-regulation of the PI-3K regulatory subunit p85 *PIK3R1*.

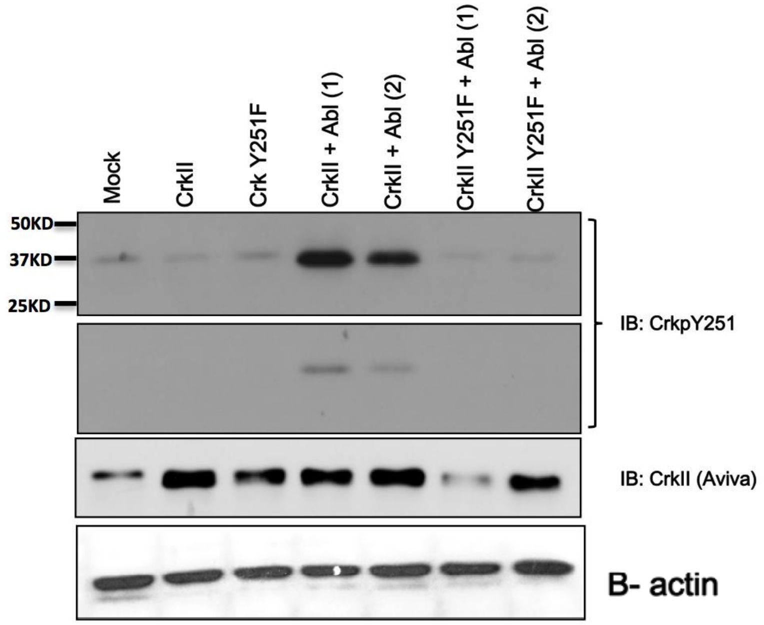

Figure S3: Characterization of CrkY251 phospho-specific polyclonal antibody: Crk-/- MEFs were transfected as shown and lysates were tested for CrkY251 phosphorylation in exogenously expressed CrkII by overexpressed Abl kinase. Total CrkII and actin were used as loading controls.

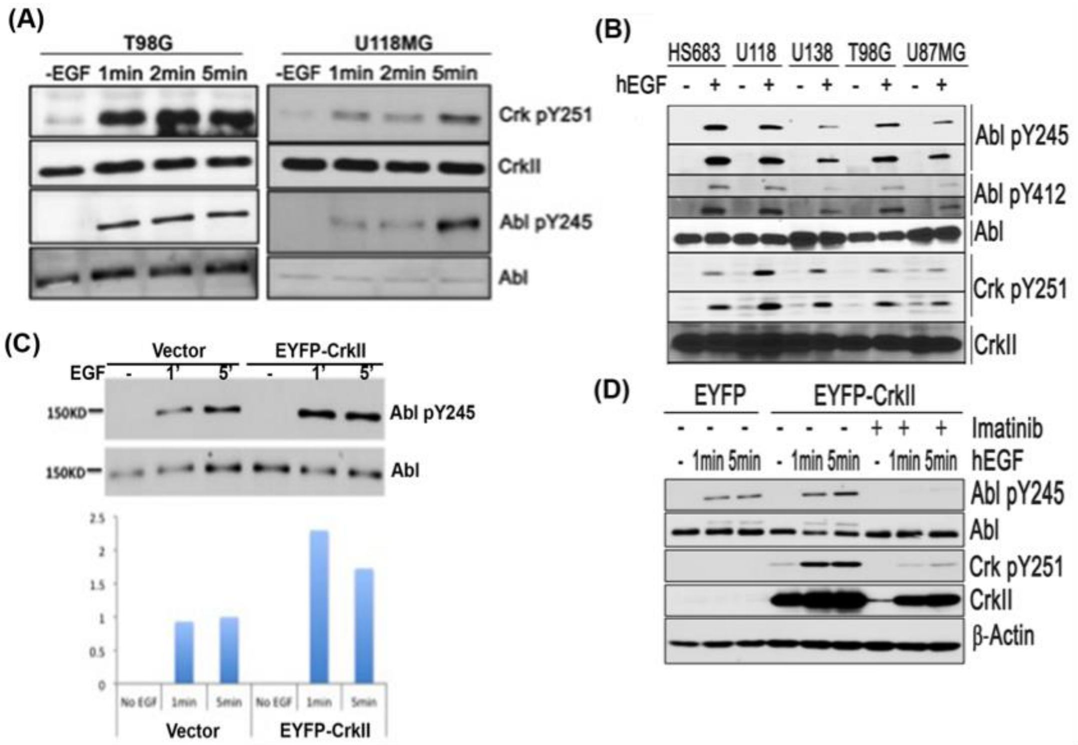

Figure. S4: **hEGF induced CrkY251 and AblY245 phosphorylation kinetics: A.** T98G and U118MG Cell lines were serum starved in 0.1% FBS containing media for 16hrs and stimulated with 100ng/ml hEGF for indicated time points and immunoblotted for CrkY251 and AblY245 phosphorylation by phosphospecific antibodies. **C-D: Upregulated CrkII-mediated Abl transactivation in HS683 cells upon hEGF stimulation:** Stable cell lines expressing EYFP or EYFP-CrkII were serum starved and stimulated for given time points. Lysates were analyzed for Abl transactivation by immunoblotting for AblY245 phosphorylation. Bar graphs shown below represent densitometry analysis of the Abl pY245 levels in the aforementioned cell lines.

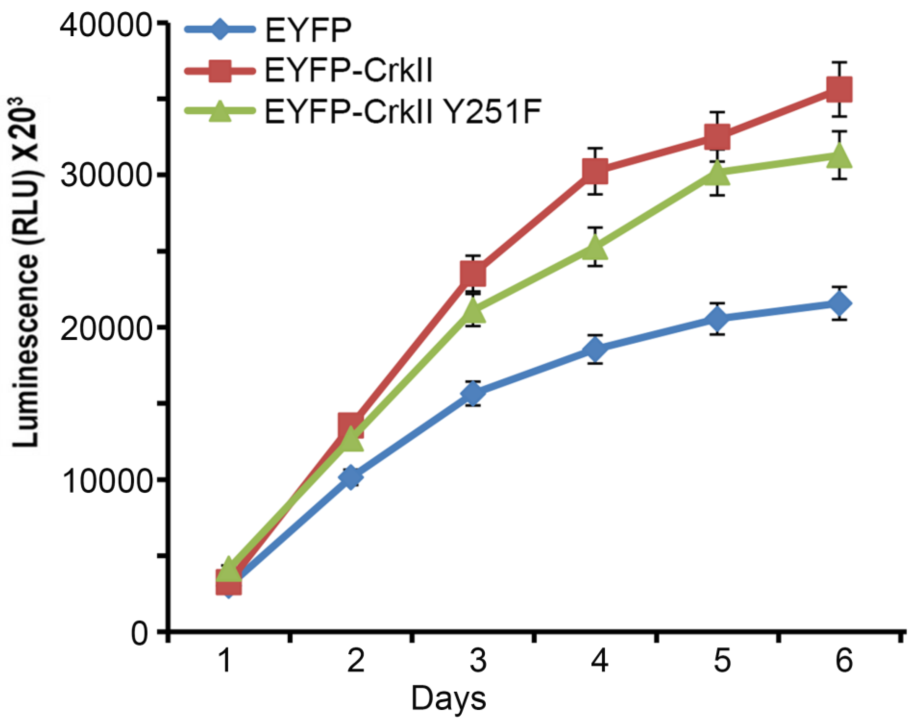

Figure S5: Comparison of cell proliferation of stable cell lines by cell viability assay: To determine the viability of HS683 stable cell lines, we used CellTiter-Glo luminescent cell viability assay kit (Promega). 20,000 cells/well in opaque walled 96-well plates containing 100µl DMEM media with 10% FBS were added. The cells were grown for the specified period and luminescent signal were measured to determine their ATP levels after the addition of CellTiter-Glo reagent as per the manufacturer's protocol. Results (normalized and shown in RLU units) indicate that CrkII mediated increase in cell proliferation is not affected significantly by CrkY251 mutation ($P>0.05$).

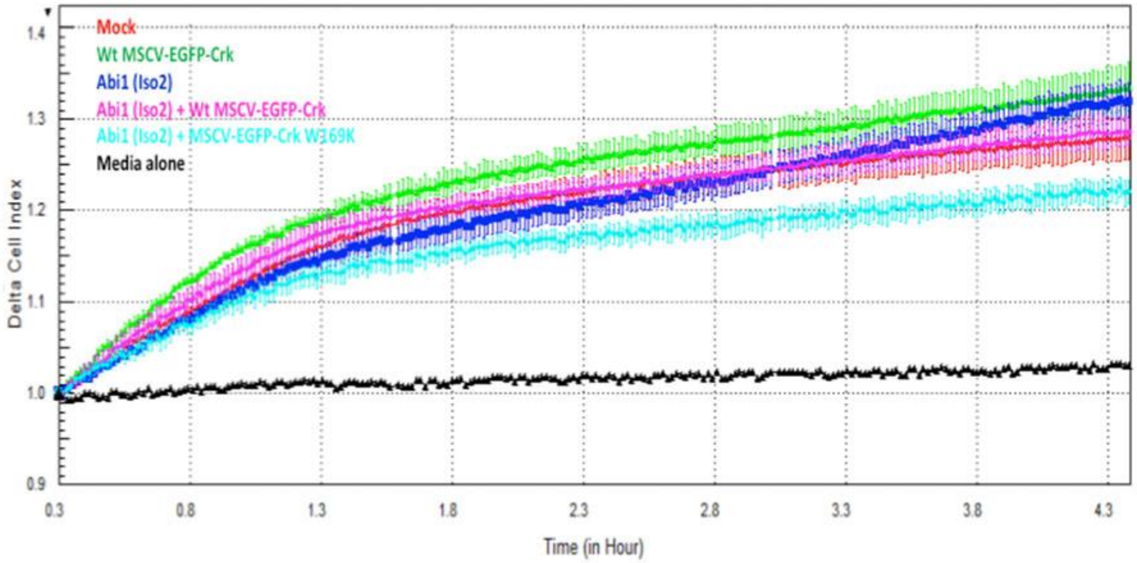

Figure S6: Real time cell spreading assay of Abi1-/- MEFs: Post-48 hours of transfection or co-transfections as shown, 40000 Abi1-/- MEFs were seeded on uncoated E-plated in triplicates and analyzed every minute for about 4 hours to study the dynamics of cell spreading by RTCA-DP real-time cell spreading instrument. Results shown here indicate that Abi1-Iso2 suppresses CrkII mediated cell spreading of Abi1-/- MEFs. This suppression in cell spreading becomes more prominent when Abi1-Iso2 is co-transfected with CrkII SH3N mutant (CrkII W169K).

Supplementary Tables

Supplementary Table 1: Association between EGFR expression and clinicopathological factors of glioma patients:

Variables	EGFR protein expression（n=43）		*P* value
	High （n=20）	Low（n=23）	
Age			**0.048**
≤47	7	15	
＞47	13	8	
Gender			0.669
Male	10	10	
Female	10	13	
Grade			**0.005**
G1-2	3	13	
G3-4	17	10	

Supplementary Table 2: Association between Abi1 expression and clinicopathological factors of glioma patients:

Variables	Abi1 protein expression（n=43）		*P* value
	High （n=26）	Low（n=17）	
Age			0.494
≤47	15	8	
＞47	11	9	
Gender			0.289
Male	11	10	
Female	15	7	
Pathological Grade			0.834
G1-G2	10	6	
G3-G4	16	11	

Supplementary methods:

Plasmids and cell culture:

For transient transfections in the mammalian cell lines pCAGGS vector encoding hCrkII and pEGFP-N1 encoding Abi1-Iso2 were used. Plasmid encoding human c-Abl 1b was provided by Dr. Giulio Superti-Furga. MSCV-EYFP-hCrkII and MSCV-EYFP were used for retroviral infections. MSCV-EYFP-Abi1-Iso2 was prepared by PCR cloning. pGEX-2T encoding GST-hCrkII was provided by Dr. Michiyuki Matsuda. pGEX 6P-1 encoding GST, pGEX-2T encoding human Abi1-Iso2 have been described previously (1, 2).
Abi1-/- and Abi1*fl/fl* MEFs were maintained in DMEM (4.5 g of glucose/l with L-glutamine) supplemented with 10%FBS and antibiotics (100 U/ml penicillin and streptomycin) at 37°C in a humidified incubator with 5% CO_2. Glioblastoma multiforme cell lines (U87, HS683, U118MG, U138MG and A172) and HEK293T cells were also maintained in the aforementioned media. Stable HS683 and Abi1-/- cell lines overexpressing human WtAbi1-Iso2, WtCrkII and CrkII Y251F were prepared by retroviral infection of respective plasmids and subsequently were selected for 7-10 days in 0.1ug/ml and 4ug/ml puromycin respectively.

Expression and purification of CrkII and Abi1-Iso2:

The recombinant clones of GST- CrkII and Abi1-Iso2 were expressed in *E. coli* BL21 and purified by affinity chromatography using glutathione Sepharose beads (Pharmacia) and Superose 6 increase 10/300 GL gel filtration columns (GE). In brief, GST- CrkII and Abi1-Iso2 transformed *E. coli* cells were grown at 37°C in LB medium with 100µg/ml ampicillin until OD_{595} was 0.5. The culture medium was cooled to room temperature, supplemented with 1mM IPTG, and further incubated at 25°C for 6h with vigorous shaking. The cell were harvested, washed with 50mM Tris-HCl containing 0.15M NaCl, and resuspended in a lysis buffer (25mM HEPES buffer, pH7.6; 0.1M KCl; 2mM EDTA; 20% glycerol; 2mM DTT; 0.1% NP40; and 1x protease inhibitors cocktail) containing 2mg/ml of lysozyme. Cells were subjected to three cycles of freezing at -80°C and

thawing, then sonicated at amplitude 40 with three 15-sec pulses. The lysed cells were centrifuged at 4°c, 15000 rpm for 20min. The supernatant was mixed with glutathione Sepharose beads (0.5mg beads/ml), incubated at 4°C for 2hrs on rotor mixture, and placed in a column, which was extensively washed with a wash buffer containing 50mM Tris-HCl (pH 8.0) and 5% glycerol. Proteins were eluted from the column with 10mM reduced glutathione (Sigma Aldrich) and further high purity fraction of proteins were loaded on Superose 6 increase 10/300 GL gel filtration columns then applied to the FPLC. CrkII and Abi1-Iso2 protein was eluted from the column using elution buffer containing (0.01M phosphate buffer, 0.14M NaCl, Ph 7.4) with 0.5ml/min flow rate. Eluted fractions showing greater than 95% purity on SDS-PAGE (8%) were dialyzed against buffer containing 50mM Tris HCl (pH 7.8), 100mM NaCl, 2mM DTT, 1mM BME, and 50% glycerol.

Cell viability assay:

To determine the viability of HS683 stable cell lines, we used CellTiter-Glo luminescent cell viability assay kit (Promega). This assay determines the number of viable cells in culture based on ATP quantitation that is directly proportional to the number of viable cells present in the culture. 20,000 cells/well in opaque walled 96-well plates containing 100ul DMEM media with 10% FBS were grown for the specified period and luminescent signal were measured to determine their ATP levels after the addition of CellTiter-Glo reagent as per the manufacturer’s protocol.
